# SHH signaling directed by two oral epithelium-specific enhancers controls tooth and oral development

**DOI:** 10.1038/s41598-017-12532-y

**Published:** 2017-10-11

**Authors:** Tomoko Sagai, Takanori Amano, Akiteru Maeno, Hiroshi Kiyonari, Hyejin Seo, Sung-Won Cho, Toshihiko Shiroishi

**Affiliations:** 1 Mammalian Genetics Laboratory, National Institute of Genetics, Mishima, Shizuoka, Japan; 2 Animal Resource Development Unit and Genetic Engineering Team, RIKEN Center for Life Science Technologies, Kobe, Hyogo, 650–0047 Japan; 3 Division of Anatomy and Developmental Biology, Department of Oral Biology, Yonsei University College of Dentistry, Seoul, Korea

## Abstract

Interaction between the epithelium and mesenchyme coordinates patterning and differentiation of oral cavity structures including teeth, palatal rugae and tongue papillae. SHH is one of the key signaling molecules for this interaction. Epithelial expression of *Shh* in the tooth buds and tongue papillae is regulated by at least two enhancers, MRCS1 and MFCS4. However, it is unclear how the two enhancers cooperate to regulate *Shh*. Here, we found that simultaneous deletion of MRCS1 and MFCS4 results in the formation of a supernumerary tooth in front of the first molar. Since deletion of either single enhancer barely affects tooth development, MRCS1 and MFCS4 evidently act in a redundant fashion. Binding motifs for WNT signaling mediators are shared by MRCS1 and MFCS4, and play a central role in regulating *Shh* expression, indicating that the two redundant enhancers additively exert their *Shh* regulation by responding to WNT signal input.

## Introduction

During development of jawed vertebrates, pairs of pharyngeal arches form many oropharyngeal apparatuses including the lip, salivary glands, teeth, tongue and palate in the mandible and maxilla. Interaction between the oral epithelium and the mesenchyme coordinates patterning and differentiation of these organs. Gene regulatory networks involving several diffusible signaling factors mediate the oral epithelium-mesenchyme interaction. The WNT and BMP signaling pathways are known as two major mediators of the interaction during early tooth development^1^. Blocking either of these pathways causes tooth developmental arrest at the dental lamina or early bud stage^1–6^. Targeted inactivation of *Lef1*, a mediator of WNT signaling, shows severe impairment in formation of teeth, as well as whiskers, hair follicles and mammary glands^7^. Conversely, over-activation of WNT signaling by expression of dominant stable *Ctnnb1* results in supernumerary teeth in the mouse^8^. These studies indicate that WNT signaling is a key factor for oral and tooth development.

SHH signaling is also an essential component of craniofacial development in vertebrates. In the oral cavity, *Shh* is expressed in epithelium of the teeth, tongue papillae and palatal rugae. Targeted disruption of *Shh* or *Smo*, which is a mediator of SHH signaling, results in fusion of molars in the mice^9,10^. Formation of supernumerary teeth adjacent to endogenous molars has been reported in mutant mice for several genes residing in the WNT and SHH signaling pathway^11–14^. A mouse mutant of *Gas1*, which is an antagonist for SHH, displays an elevated SHH activity and results in a supernumerary tooth in the diastema, which is a gap between the incisors and molars^11^. In contrast, knockdown of SHH signaling by applying an anti-SHH antibody shows molar fusion or supernumerary tooth formation in mouse embryos^13^. Therefore, both of upregulation and downregulation of SHH signaling potentially cause extra tooth. A mouse mutant of *Sostdc1* (*Wise*, *ectodin*, *USAG-1*), which is downstream of *Shh* and is a secreted antagonist of WNT and BMP signaling, also exhibited supernumerary molars, probably due to elevated WNT signaling^12,13^. On the other hand, inhibition of WNT signaling results in downregulation of *Shh* in tooth buds^15^. These studies imply that *Shh* is a downstream target of WNT signaling, and that, in turn, it inhibits WNT signaling via a negative feedback loop between the SHH and WNT signaling pathways. Taken together, these observations indicate that SHH is a crucial modulator for WNT signaling to maintain the proper number and normal shape of teeth. However, mechanisms controlling the expression level of *Shh* are not completely understood.

Spatiotemporal expression of *Shh* is regulated by tissue-specific enhancers located within the 1 Mb upstream of the *Shh* transcription start site (TSS)^16–21^. Among them, MRCS1, MFCS4 and MACS1 roughly partition *Shh* expression in the gastrointestinal tract into three domains of the mouth, pharynx and gut. In the oral epithelium, MRCS1 directs *Shh* expression in the secondary palate, tooth and tongue papillae. MFCS4 has regulatory activity in the soft palate, epiglottis and tooth rudiments. The expression domains of these two enhancers overlap in some oral tissue including the tooth buds^21^.

In this study, to illuminate how the two enhancers, MRCS1 and MFCS4, contribute to *Shh* regulation in the oral epithelium, we generated a double knockout (KO) mouse for MRCS1 and MFCS4. We found that simultaneous loss of the two enhancers causes a reproducible supernumerary tooth. Because deletion of a single enhancer had little or almost no effect on oral development, this result clearly indicated that the two enhancers have redundant and cooperating functions for *Shh* regulation and oral morphogenesis. Furthermore, we show that mouse MRCS1, but not MFCS4, induces a reporter expression pattern similar to that induced by *Xenopus* MFCS4, which has little sequence similarity to mouse MRCS1 except for some motifs for the transcription factor TCF/LEF. This result suggests that MRCS1 and MFCS4 direct *Shh* expression in the oral epithelium by responding to WNT signaling, and that mouse MRCS1 rather than MFCS4 has taken over the function of the ancestral MFCS4 during mammalian evolution.

## Results

### Loss of MRCS1 causes a supernumerary tooth with low frequency

**Figure 1:**
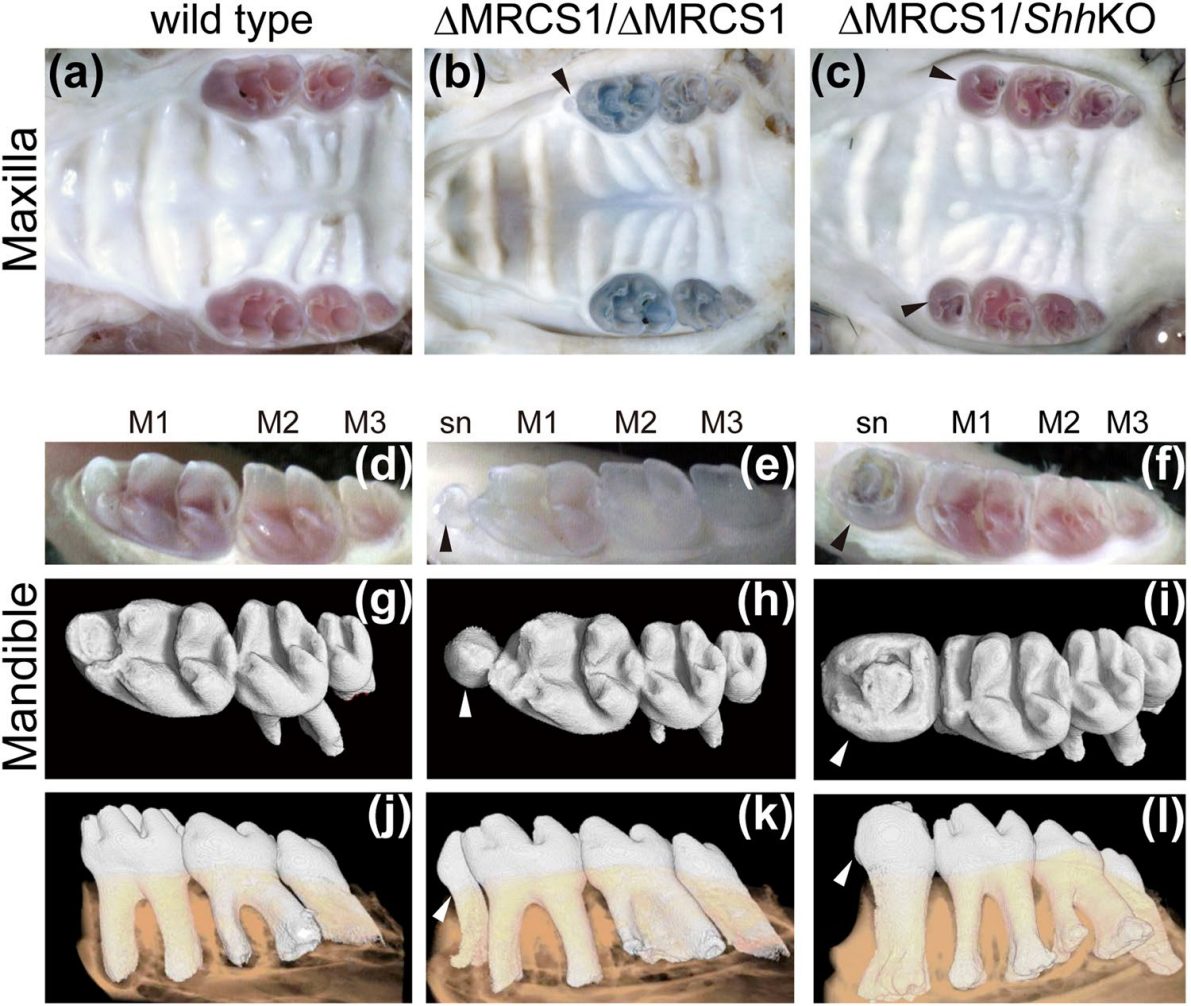
Molar pattern in the MRCS1 KO homozygote and the compound heterozygote of MRCS1 and *Shh* KO alleles. Occlusal views of maxilla in the wild type mouse (**a**), the MRCS1 KO homozygote (**b**, ΔMRCS1/ΔMRCS1) and the compound heterozygote (c, ΔMRCS1/ShhKO) at one month old. Molar pattern in the mandible of the wild type (**d**,**g**,**j**), the MRCS1 KO homozygote (**e**,**h**,**k**) and the compound heterozygote (**f**,**i**,**l**). Three-dimensional reconstructions of X-ray micro CT images of molars are shown in **g–l**. Arrowheads mark supernumerary teeth. The alleles of the compound heterozygote of the ΔMRCS1 and the *Shh* coding sequence KO alleles are schematically illustrated in Supplementary Fig. 1d. M1, first molar; M2, second molar; M3, third molar.

To test the endogenous role of MRCS1, we eliminated a 673-bp stretch of mouse genomic DNA, which is sufficient for the enhancer activity of MRCS1, by standard ES cell targeting (Supplementary Fig. 1a). The wild type mouse has three molars in each jaw quadrant (Fig. 1a,d,g,j). Despite driving strong expression in the oral tissue in a transgenic reporter assay, the MRCS1 KO homozygous mice scarcely showed a visible phenotype in the oral tissues. Of 21 mice, only one exhibited a small supernumerary tooth adjacent to the first molar (M1) in three of four jaw quadrants (Fig. 1b,e,h,k). The tooth is very small, but it has a tooth root independent of M1 (Fig. 1k). To test whether formation of the supernumerary tooth in the MRCS1 KO homozygote is relevant to *Shh* regulation, we generated compound heterozygotes by cross mating of heterozygotes of the MRCS1 KO and *Shh* coding sequence KO mice, and examined the tooth phenotype (Supplementary Fig. 1d). Notably, about 80% of the compound heterozygotes possessed a supernumerary tooth in each jaw quadrant (Fig. 1c,f,i,l and Supplementary Table 1). Even though the sizes of these supernumerary teeth are variable in the compound heterozygotes, their localized position is consistently in front of M1 (Fig. 1c,f,i,l). Since single heterozygotes of either *Shh* coding sequence KO or MRCS1 KO mice have no defect in tooth development including formation of supernumerary teeth (Supplementary Table 1), the result clearly indicates that the observed tooth defect depends on SHH signaling, and that level of SHH signaling activity is crucial for formation of the supernumerary tooth.

### Simultaneous loss of MRCS1 and MFCS4 raises incidence of a supernumerary tooth

**Figure 2:**
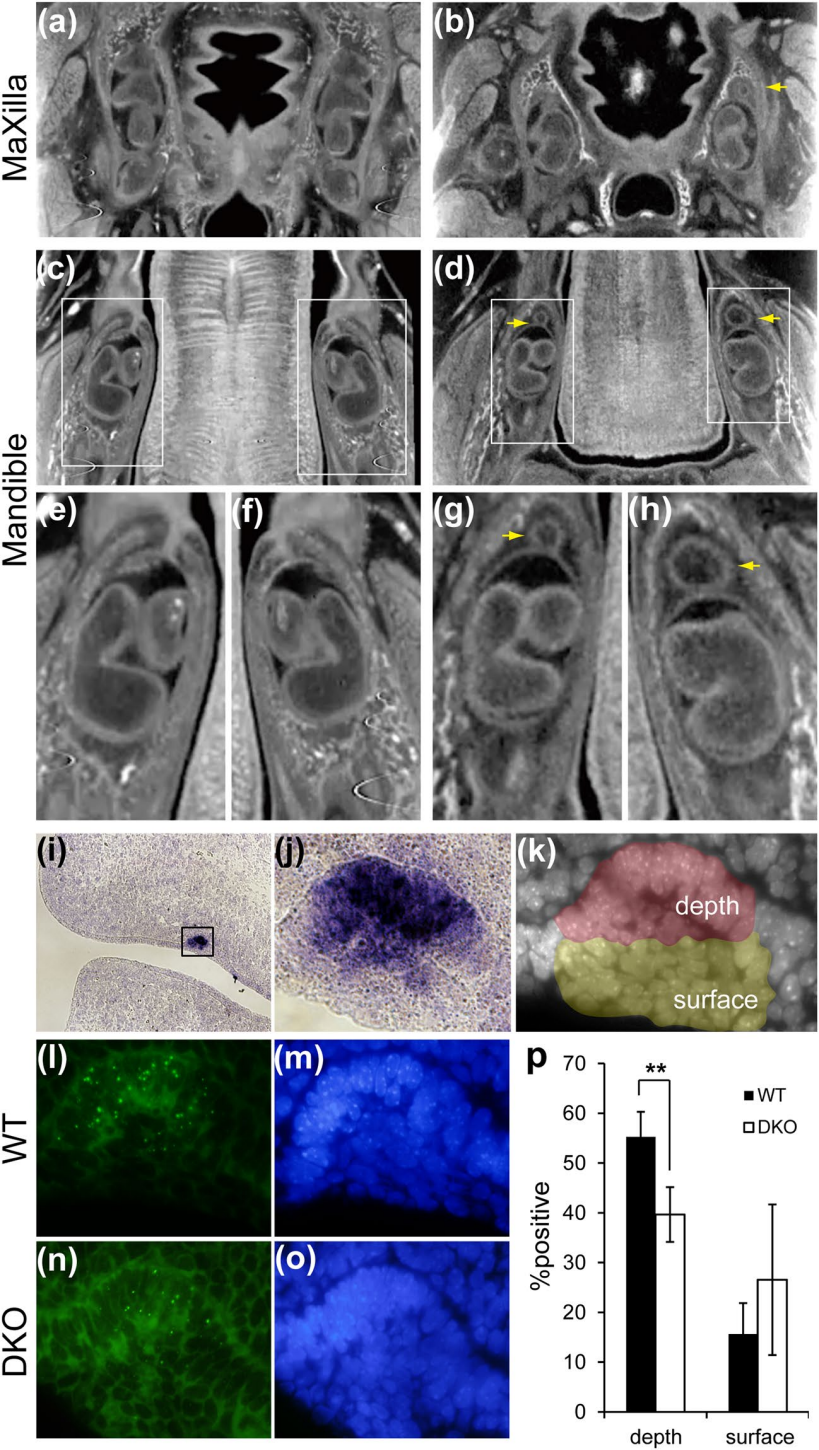
Formation of the supernumerary tooth in the double KO homozygote of MRCS1 and MFCS4. Transverse sectional micro-CT images of the maxilla (**a**,**b**) and mandible (**c**–**h**) in the wild type (WT) (**a**,**c**,**e**,**f**) and the DKO mouse (**b**,**d**,**g**,**h**). Maxillary tooth in the WT (**a**) and the DKO mouse (**b**). Mandibular tooth in the WT (**c,e,f**) and the DKO mouse (**d,g,h**). Magnified pictures of the insets of c (**e**,**f**) and of d (**g**,**h**). Yellow arrows indicate supernumerary teeth. The double KO homozygote of MRCS1 and MFCS4 is schematically illustrated in Supplementary Fig. 1e. Expression of *Shh* mRNA in the dental placode of the WT at E12.5 (**i**) and high magnification of the dental placode (**j**, an open box in **i**). Nuclei of the dental placode were stained with DAPI. The depth half and the superficial half are shown in red and yellow, respectively (**k**). Signals for *Shh* nascent RNA in the dental placode of the WT and DKO (**l,n**), and cells were stained with DAPI (**m,o**). The %positive cells transcribing *Shh* in the depth and the surface of the dental placode (**p**). The black and open bars depict the wild type and the DKO, respectively. Error bars represent the standard deviations obtained from three independent samples. Two-tailed Student’s t-test was used to test significance of differences in number of signal-positive cells (**P < 0.001).

In our previous study, a mouse transgenic reporter assay showed that another enhancer named MFCS4 also drives reporter expression in tooth buds^21^. Therefore, we hypothesized that MRCS1 and MFCS4 have redundant roles for *Shh* regulation in the tooth buds, and that they additively control the level of SHH signaling activity. To test this hypothesis, we generated double KO (DKO) mice for MRCS1 and MFCS4 (for details, see Methods and Supplementary Fig. 1e), and observed the phenotype of mice homozygous for both KO alleles. We found that the DKO homozygotes died soon after birth due to impairment of the respiratory organs, which was most likely caused by the loss of MFCS4^21^. Therefore, we examined the tooth phenotype of DKO homozygotes at E18.5 using X-ray micro-CT. They exhibited formation of a supernumerary tooth, which was also observed in the MRCS1 single KO mouse and in compound heterozygotes of the *Shh* coding sequence KO and MRCS1 KO alleles (Fig. 2a–h). The frequency of a supernumerary tooth in at least one jaw quadrant was 67% (8/12) (Fig. 2b,d,g,h), which is markedly higher than that observed in each single KO homozygote (Supplementary Table 1). Considering that the loss of either MRCS1 or MFCS4 alone has little effect on tooth development, this result indicated that the two enhancers, MRCS1 and MFCS4, control tooth morphogenesis in a cooperative manner.

In the early phase of the tooth bud formation, the oral epithelium forms a hypertrophic structure called as the dental lamina. *Shh* is intensely expressed in the depth of the dental lamina at E12.5^22^ (Fig. 2i,j). In order to compare a small difference in the expression level of *Shh* between the wild type and DKO mice, we detected nuclear nascent RNA of *Shh* rather than cytoplasmic steady-state mRNA. Dotted signals of nascent RNA were frequently observed in the deep half region in which *Shh* is intensely expressed (Fig. 2k–o). While more than half (~55%) of cells in the depth half region of the wild type were positive for the *Shh* nascent RNA, the nascent RNA positive cells in the same region were significantly reduced to 40% in the DKO mouse (Fig. 2p) In the superficial half region, frequency of the nascent RNA positive cells was not significantly different between the wild type and DKO mice. These results showed that additive action of MRCS1 and MFCS4 are necessary for precise control of the expression level of *Shh*, although these enhancers may be dispensable for the initiation of *Shh* expression in the tooth buds.

### MRCS1 and MFCS4 share common regulatory motifs

**Figure 3:**
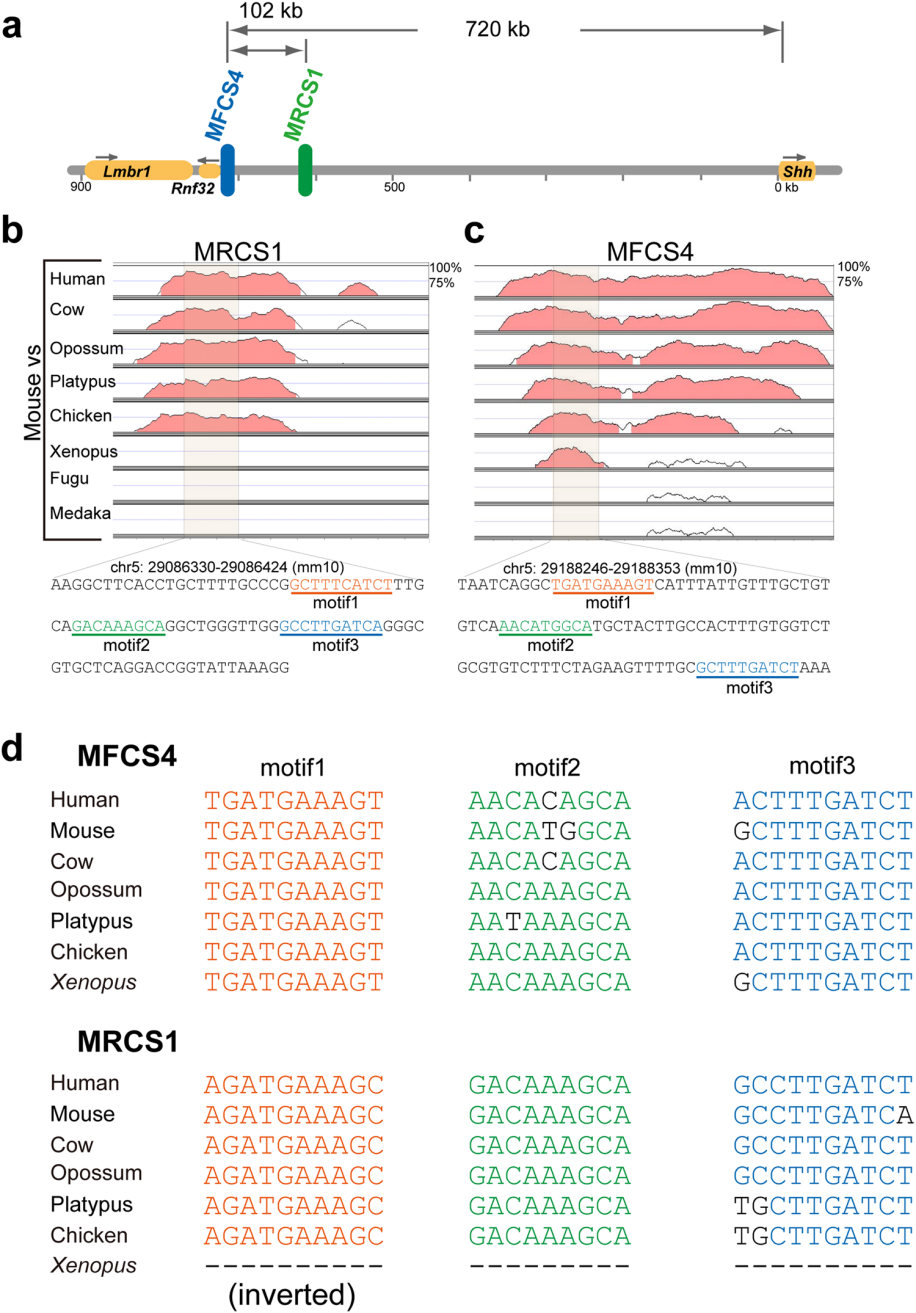
Motifs shared between MRCS1 and MFCS4. (**a**) Diagram of the upstream region of the mouse *Shh* locus. The two epithelial enhancers MFCS4 (blue) and MRCS1 (green) are located at 620–720 kb upstream of the *Shh* TSS. Comparison of mouse MRCS1 (**b**) and MFCS4 (**c**) in vertebrate taxa by Vista plots. A shaded region represents the core unit of the enhancer, which contains three shared motifs. The core sequences are shown at the bottom of (**b**). (**d**) Alignments of the three motifs in the MFCS4 and MRCS1 sequences, which were obtained from seven vertebrate species.

MRCS1 and MFCS4 are evolutionarily conserved enhancers, which are located close to each other at a long distance from the *Shh* coding sequence (Fig. 3a). The evolutionary conservation ranges of the two enhancers are different. While MFCS4 is conserved across all vertebrates (from mammals to fish), conservation of MRCS1 ranges from mammals to reptiles^21^ (Fig. 3b,c). To elucidate whether MRCS1 and MFCS4 share similar or identical short sequences for *Shh* regulation, we compared the most conserved region of MRCS1 and MFCS4 of two mammalian species, mouse and opossum. As a result, the most conserved regions of two oral enhancers shared two types of short sequence. The shared sequence 1 overlapped consensus motifs for binding of TCF/LEF proteins (motif1 and motif3, Supplementary Fig. 2a). The shared sequence 2 contains a putative binding site for SOX and MEIS proteins (motif2). The three motifs are well conserved among remote taxa of vertebrates, and occur in the same order in MRCS1 and MFCS4 (Fig. 3b,c,d). There is no marked similarity in sequence outside of these three motifs between the two enhancers. Hereafter, we refer to these three common motifs as a core unit. To examine whether the core unit functions alone in the oral epithelium, we generated transgenic mice with a *LacZ* reporter construct containing three tandem copies of the 95-bp core sequence (chr5: 29086330–29086424, GRCm38/mm10) of the mouse MRCS1 (Supplementary Fig. 2b). Reporter signals were observed in the tooth and tongue papillae, but not in the palatal rugae (Supplementary Fig. 2c). TCF/LEF proteins, which mediate WNT/CTNNB signaling, play a crucial role in tooth development^23^ Several members of the Sox protein family, which is known to regulate WNT signaling, are also necessary for tooth development^24^. Evolutionarily conserved motifs for TCF/LEF may be involved in possible crosstalk between the SHH and WNT signaling pathways.

### A LEF1 binding motif is essential for the enhancer activity of MRCS1

**Figure 4:**
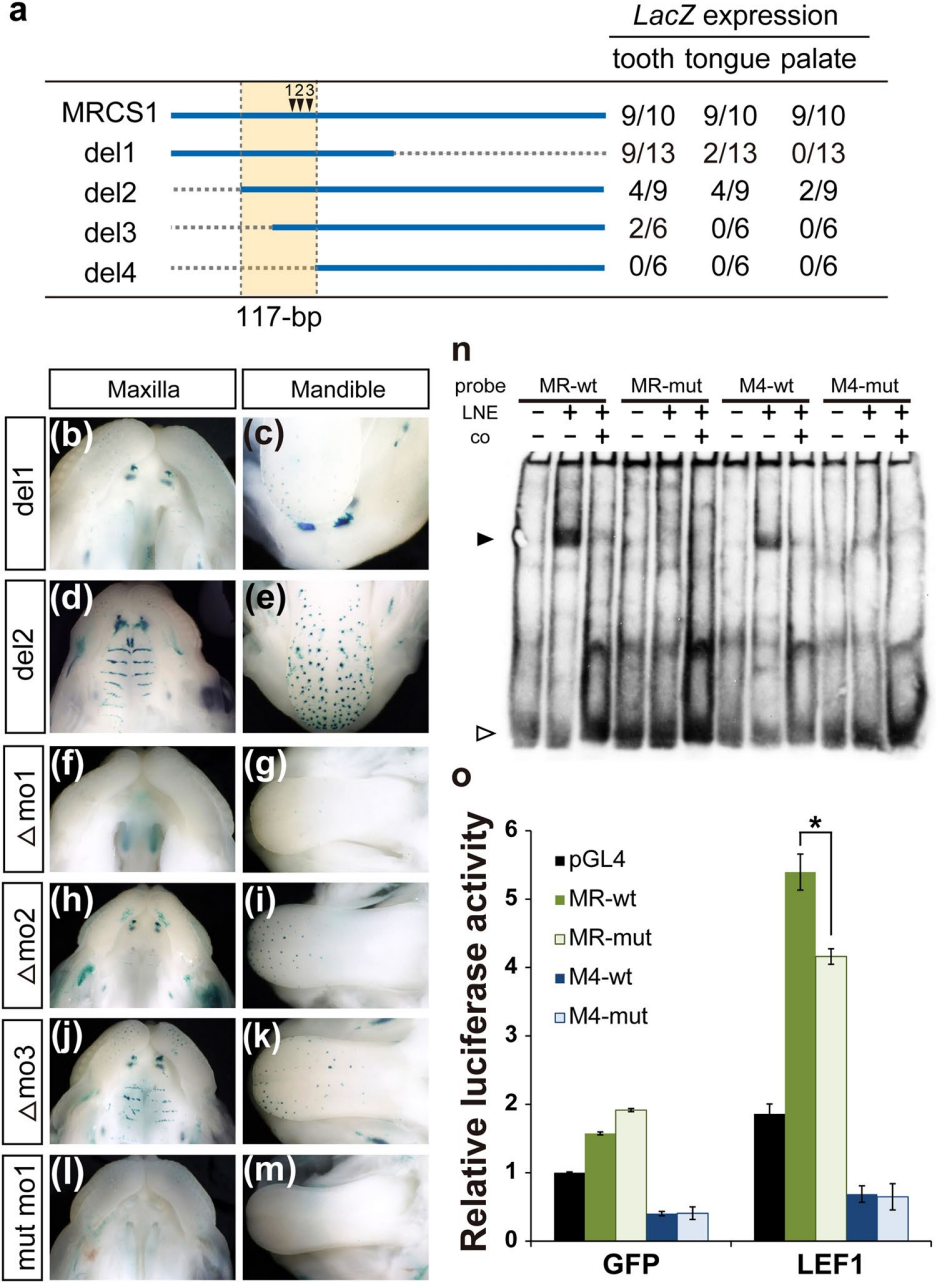
*LacZ* expression patterns in transgenic mice with MRCS1 deletion constructs. Diagram of deletion constructs of MRCS1 (**a**). Dotted lines show the region deleted in each construct. Arrowheads indicate the three motifs shown in Fig. 3. Numbers of *LacZ*-positive embryos among transgene-positive embryos in each organ are shown in the right column. *LacZ* expression patterns in the maxilla (**b**,**d**) and mandible (**c**,**e**) with deletion construct 1 (del1; **b**,**c**) and deletion construct 2 (del2; **d**,**e**). *LacZ* expression patterns in transgenic mice with deletions of motif1 (Δmo1: **f,g**; n = 6), motif2 (Δmo2: **h,I**; n = 8) and motif3 (Δmo3: **j,k**; n = 7) and a silent mutation of motif1 (mut mo1: **l,m**; n = 10). (**n**) EMSA with sequences at motif1 of MRCS1 and MFCS4 (MR-wt and M4-wt), and sequences with a silent mutation of motif1 (MR-mut and M4-mut, respectively). An arrowhead indicates a specific band shift. An open arrowhead indicates free probes. LNE is an abbreviation of nuclear extracts derived from *Lef1*-overexpressing cells. Cold oligos as a competitor, co. (**o**) Relative luciferase activity of the core MRCS1 (MR-wt), the core MRCS1 with a silent mutation of motif1 (MR-mut), the core MFCS4 (M4-wt) and the core MFCS4 with a silent mutation of motif1 (M4-mut) in HEK293T cells. The luciferase reporter constructs containing the core enhancer sequences were cotransfected with plasmid vectors expressing *Gfp* and *Lef1*. The reference value for cotransfection with *Gfp*-expressing and pGL4 empty plasmids was set as 1. Error bars represent the standard deviations obtained from three independent experiments. An asterisk shows a significant difference, as evaluated by Student’s t test (**o**, p < 0.05).

Three tandem repeats of the core sequence of MRCS1 induced *LacZ* reporter expression in the mouse dental lamina and tongue papillae. The expression domain almost completely overlapped that induced by a 710-bp fragment containing the whole mouse MRCS1 sequence. On the other hand, palatal expression was not induced by the 95-bp core sequence of MRCS1. Thus, the core unit of MRCS1 alone does not exert the entire regulatory activity of MRCS1. To dissect the regulatory function of the whole MRCS1 sequence, we generated a series of deletion constructs from the whole MRCS1 sequence (Fig. 4a), and carried out transgenic reporter assays. The 5′ half of MRCS1 (del1) induced reporter expression in the tooth and tongue papillae, but not in the palatal rugae (Fig. 4b,c). Deletion of a 100-bp region from the 5′ end of MRCS1 (del2) did not strongly affect the *LacZ* expression pattern in the tooth and tongue papillae, or in the palatal rugae (Fig. 4d,e). These results suggest that the 3′ region of MRCS1 contains an element that regulates palatal expression. A series of 5′ deletion constructs (del2–4) revealed that a 117-bp sequence is indispensable for enhancer activity in the oral epithelium. This region contains the 95-bp core sequence of MRCS1, which includes three evolutionarily conserved motifs (Figs 3 b,4a).

To confirm involvement of the WNT signaling pathway in *Shh* regulation, we abolished each of the three motifs in the core unit from the mouse MRCS1 sequence. Deletion of motif1 abrogated reporter expression in the MRCS1-*LacZ* transgenic mouse (Fig. 4f,g). Deletion of motif2 diminished expression in the palatal rugae and in part of the tongue papillae, with *LacZ* expression remaining in the tooth buds (Fig. 4h,i). Deletion of motif3 moderately altered the *LacZ* expression patterns in the palatal rugae and tongue papillae (Fig. 4j,k). Motif1, which binds to a TCF/LEF protein, plays a pivotal role for MRCS1 activity. The requirement of this motif for gene regulation was further confirmed by silent substitution that abrogated the TCF/LEF binding (Fig. 4l,m).

To assess if TCF/LEF family proteins can bind to the oral enhancers, we performed an electrophoretic mobility shift assay (EMSA) using nuclear extracts derived from cells expressing *Lef1*. Probes containing motif1 of MRCS1 and MFCS4 demonstrated a specific band shift in the presence of nuclear extract (Fig. 4n). This band shift was markedly prevented by either the silent mutation at motif1 or adding a competitor cold oligo. This result suggested that LEF1 potentially binds to motif1 of both MRCS1 and MFCS4.

To test the effect of LEF1 binding to motif1 in mammalian cells, we conducted a luciferase reporter assay with core sequences of MRCS1 and MFCS4. Cotransfection of a *Lef1* expression construct with the MRCS1 luciferase reporter resulted in elevated expression of luciferase in HEK293T cells as compared with transfection of a *Gfp*-expressing control plasmid (Fig. 4o). The silent mutation at motif1 of MRCS1 showed reduced luciferase activity. In contrast, the core MFCS4 did not show significant activation of the reporter even with cotransfection of the *Lef1* expression construct. Binding of LEF1 to MFCS4 may be insufficient to upregulate *Shh* expression. Thus, motif1 of MRCS1 and MFCS4 are functionally different, although both motif1 potentially bind to LEF1.

### Functional similarity between mouse MRCS1 and Xenopus MFCS4

**Figure 5:**
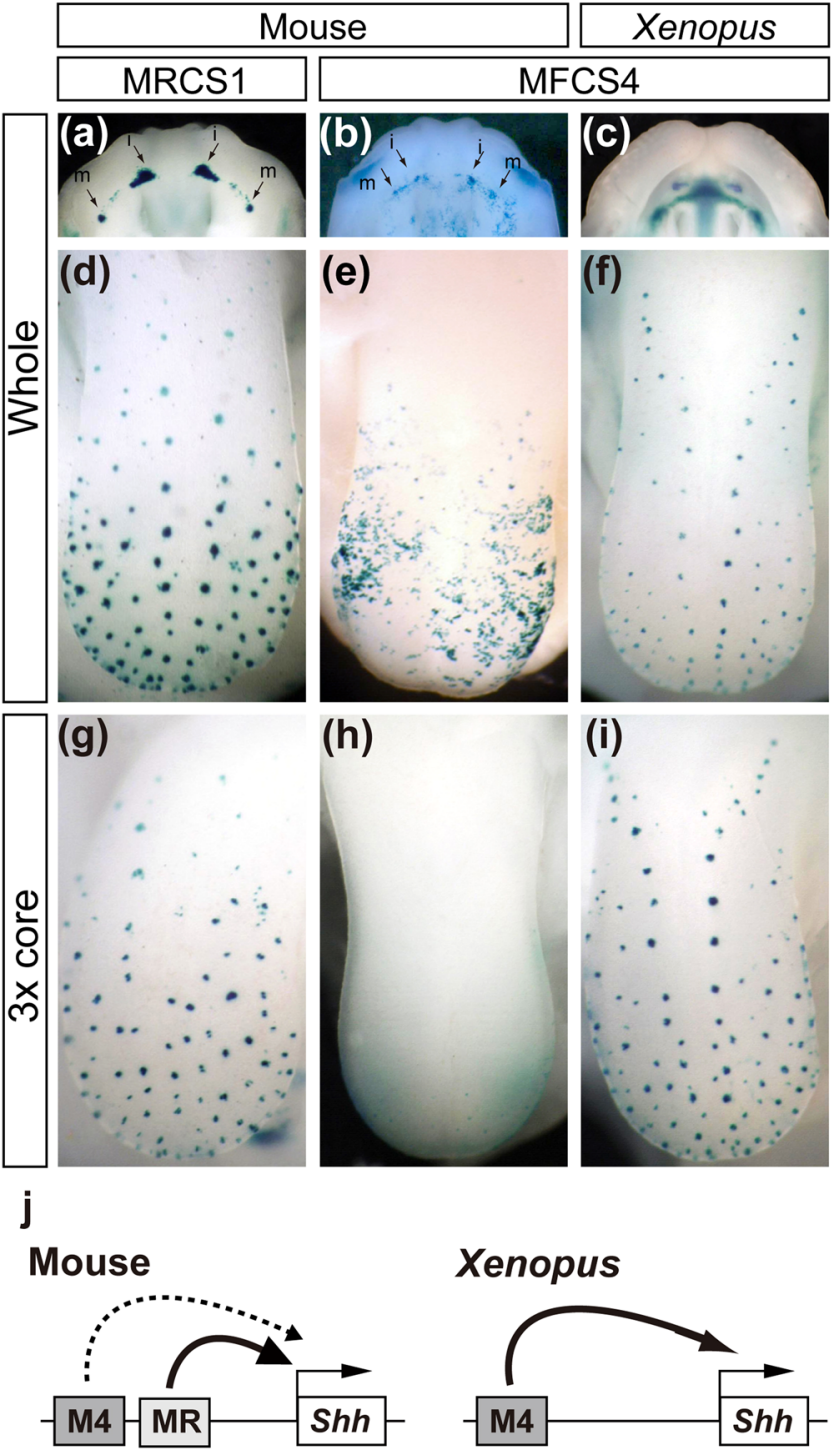
Functional similarity between mouse MRCS1 and *Xenopus* MFCS4. *LacZ* expression in the teeth buds of the maxilla (**a**–**c**) and papillae of the tongue (**d**–**i**). Expression directed by the whole fragments of mouse MRCS1 (**a**,**d**), mouse MFCS4 (**b**,**e**), *Xenopus* MFCS4 (**c**,**f**) or three tandem copies of the core sequence of mouse MRCS1 (**g**), mouse MFCS4 (**h**) or *Xenopus* MFCS4 (**i**). (**j**) Schematic diagram of *Shh* regulation by MRCS1 (MR) and MFCS4 (M4). MRCS1 primarily regulates *Shh* in the oral epithelium of mouse. MFCS4 alone regulates *Shh* in *Xenopus*.

MRCS1 and MFCS4 are functionally redundant in the tooth and tongue papillae of the mouse embryo. Compared with mouse MRCS1, mouse MFCS4 induced weak reporter signals in the incisor and molar rudiments (Fig. 5a,b). Reporter expression in the dental lamina induced by mouse MFCS4 begins at E11.5 and terminates earlier than that induced by MRCS1, suggesting that MFCS4 is involved in tooth formation for a shorter period during development. Moreover, the reporter expression induced by mouse MFCS4 was non-discrete in the tongue, and was not specific to the spotted tongue papillae (Fig. 5d,e). Given that MFCS4 induced weak, short-term and less-specific expression in the mouse oral epithelium, MRCS1 but not MFCS4 is likely a primary enhancer for *Shh* in mouse oral cavity development. The difference in strength of the reporter expression implies sub-functionalization of regulatory activity between the two redundant enhancers.

The *Xenopus* genome has a sequence orthologous to mouse MFCS4; it shares the core unit containing the three motifs, but shows no overt similarity to the mouse MRCS1 sequence. To test the possibility that the *Xenopus* MFCS4 directs *Shh* expression in oral tissues, we obtained an MFCS4 ortholog from the *Xenopus* genome, and carried out a reporter assay with the *Xenopus* MFCS4 sequence in transgenic mice. *Xenopus* MFCS4 indeed induced *LacZ* reporter expression in the tooth and tongue papillae (Fig. 5c,f). Notably, the *LacZ* reporter signals were intense, and the expression pattern was similar to that induced by mouse MRCS1, but not mouse MFCS4. In particular, three tandem copies of the core sequence of the *Xenopus* MFCS4 induced the *LacZ* expression pattern specifically in the tongue papillae, which is similar to that induced by the tandem copy of the mouse MRCS1 (Fig. 5g,i). By contrast, tandem copies of core sequences of mouse MFCS4 did not induce such an expression pattern (Fig. 5h). This result suggests that MRCS1 and MFCS4 are sub-functionalized in mouse, and that MRCS1 is a functional successor of the ancestral MFCS4 (Fig. 5j).

## Discussion

Orchestration of gene regulatory networks is essential for normal organogenesis. Developmental genes are generally regulated by multiple enhancers that confer similar expression domains in tissues. Therefore, disruption of a single enhancer often causes no morphological abnormality in animal organs. In the present study, we found that the incidence of a morphological defect in mouse teeth significantly increased when MRCS1 and MFCS4, two oral enhancers of *Shh*, were simultaneously disrupted, indicating that redundant activities of these two enhancers contribute to the normal number and shape of teeth. Given that deletion of either one of the two enhancers somewhat downregulated *Shh* expression, additive effects of the two enhancers are crucial for *Shh* regulation. Perhaps, when the level of *Shh* expression drops below a certain threshold value, a supernumerary tooth appears as a morphological abnormality. Similar regulation by multiple enhancers has been reported for other developmental genes such as *Prx* and *Hoxd* ^25^. Redundant enhancers contribute to fine-tuning of gene expression levels, and thereby ensure normal organogenesis. In *Drosophila*, such redundant enhancers account for the robustness of gene expression against environmental fluctuations such as a change of temperature^26^.

There was a large difference in the intensity and duration of *LacZ* reporter expression in the transgenic mice between the MRCS1 and MFCS4 constructs. Lower intensity and shorter duration were observed in reporter expression directed by MFCS4, as compared with MRCS1. MFCS4 appears to act as a secondary enhancer - in other words, a shadow enhancer - in the oral epithelium. Complete loss of *Shh* from the dental lamina in a conditional KO mouse reportedly causes a fused molar in each quadrant^9^, which is different from the phenotype observed here in homozygous mice with the double KO of the two enhancers. Therefore, yet another enhancer is most likely required for full *Shh* expression in oral tissues to ensure the robustness of tooth development.

It is now generally accepted that *cis-*regulatory elements of pleiotropic developmental genes contributed more than coding sequences to morphological evolution of vertebrates^27–33^. *Shh* expression in mesenchymal cells, which constitute the zone of polarizing activity in the limb buds, is regulated by a single enhancer named MFCS1 (also known as ZRS)^17,34^. Removal of MFCS1 from the mouse genome results in the loss of distal limb structures^34^. Progressive changes in the sequence of the limb enhancer are relevant to limb loss in snake evolution^33,35^; this is a clear instance of an enhancer acting as a possible source of morphological evolution, although it remains elusive why the regulation of *Shh* for limb development is controlled by only one enhancer. Another example was recently presented by our study in which morphological transition from ventral lungs to dorsal gas bladder in the teleost fish lineage was associated with changes of endodermal epithelium-specific *Shh* enhancers^36^. Redundant enhancers may be advantageous in that they give rise to sequential and functional variations with little evolutionary constraint. Indeed, our data indicate that sub-functionalization of regulatory activity for *Shh* expression occurs between MRCS1 and MFCS4 in the mouse. Mouse MFCS4 mainly regulates *Shh* expression in the pharynx, with relatively weak enhancer activity in the oral epithelium^21^. Thus, the *Shh* regulation in oropharyngeal domains is subdivided into MRCS1-driven regulation for the oral epithelium and MFCS4-driven regulation for the pharyngeal epithelium.

In mammalian evolution, the tooth number has been reduced from ancestral forms^37^. In the mouse, there are only one incisor and three molars in each jaw quadrant. During mouse embryogenesis, some vestigial teeth are transiently formed in the diastema between the incisor and the first molar. Vestigial teeth regress by apoptosis, and are incorporated into an anterior part of the first molar in the mandible at a later developmental stage. Previous analysis using 3D-reconstructed images of molars and molecular markers revealed that mouse embryos have two rudimentary buds, MS and R2, which exhibit transient *Shh* expression in front of the first molar^37^. Since mutations that affect oral expression of developmental genes including *Shh* and *Wnt* cause a supernumerary tooth^11–14^, disappearance of rudimentary tooth buds is a key process of tooth development under the control of major signaling pathways. The mechanisms by which the R2 bud is incorporated into the first molar can account for the loss of the premolar during mouse evolution^14,37–41^. In this context, the phenotype observed in mice having deletions of the oral epithelium enhancers of *Shh* may be atavistic.

The WNT-SHH negative feedback loop is known as a pivotal mechanism controlling the spatial patterning of teeth^12,13^. Inhibition of WNT signaling by *Sostdc1*, which is positively regulated by *Shh*, is involved in the regulation of tooth number and shape. Multiple supernumerary teeth develop in *K14-Cre*; *Ctnnb1* ^*(Ex3)fl/*+^ and *K14-Cre*; *APC* ^*cko/cko*^ mice^42,43^ as a result of sustained WNT signal activation in the epithelium. Molar fusion has been reported only in *K14-Cre*; *Shh* ^*fl/fl*^ mice, *K14-Cre*; *Smo* ^*fl/fl*^ mice, *Sostdc1* ^−/−^ and *Lrp4* ^−/−^ mice^9,12,44,45^. *Sostdc1* ^+/−^; *Shh* ^+/−^ mice show only a few supernumerary teeth, but *Sostdc1* ^+/−^ mice and *Shh* ^+/−^ mice do not show any phenotype^12^. These previous studies indicated that magnitude of the tooth phenotype is proportional to the extent of the deficiency in SHH and SOSTDC1 signals, and the abundance in WNT signals. This dependence of phenotypic change on the level of gene expression is consistent with our finding that the extent of *Shh* enhancer loss is proportional to the incidence of supernumerary teeth. On a smaller scale, phenotypic change can also be triggered by minor variations in enhancer DNA sequence. Deletion and silent substitution of a TCF/LEF binding motif in MRCS1 markedly abrogated reporter expression in the epithelium of tooth buds, palatal rugae and tongue papillae, suggesting that a TCF/LEF binding motif is necessary for regulatory activity of the oral *Shh* enhancer MRCS1. This finding supports the assertion that there is a direct link between SHH and WNT signaling and that morphological changes can be caused by only a few differences in the specific DNA sequence of an enhancer.

## Methods

### Mice

Mice were purchased from CLEA Japan (Tokyo, Japan). The *Shh* KO mutant, which was a kind gift from Dr. P. Beachy, is maintained in the C57BL/6 background in the National Institute of Genetics (NIG). All animal experiments in this study were approved by the Animal Care and Use Committee of NIG and Institutional Animal Care and Use Committee of RIKEN Kobe Branch. All methods were performed in accordance with the approved guidelines and regulations of NIG and RIKEN Kobe Branch.

### Comparative analysis

Genomic sequences of vertebrates were obtained from the UCSC genome browser (http://genome.ucsc.edu/)^46^. For sequence comparison and visualization, VISTA (http://genome.lbl.gov/vista/mvista/submit.shtml)^47^ was used. Local common sequences in enhancers (shared sequences) were identified by MEME (http://meme-suite.org/)^48^. Potential TF-binding motifs in/around the shared sequences were surveyed by JASPAR (http://jaspar.genereg.net/)^49^. Sequence alignment was generated by the ClustalW program (http://clustalw.ddbj.nig.ac.jp/).

### ESC targeting

The MRCS1 KO mouse strain was generated in RIKEN (Accession No. CDB0781K: http://www2.clst.riken.jp/arg/mutant%20mice%20list.html). Primer pairs used for vector construction and genotyping are listed in Supplementary Table 2. We used pKO Scrambler V901 as a targeting vector by inserting a floxed neomycin cassette and a DT-A negative selection marker (Supplementary Fig. 1a)^34^. The long and short arms were PCR-amplified from RP23-284A9 BAC DNA and cloned into the targeting vector. A 673-bp (chr5: 29085875-29086547, GRCm38/mm10) fragment including mouse MRCS1 was replaced with the floxed neomycin cassette by homologous recombination in TT2 ES cells^50^. Correct homologous recombination was confirmed by Southern blot analysis (Supplementary Fig. 1b). Chimeras were mated with C57BL/6 for germline transmission. The neomycin cassette in the targeted mouse was removed by mating with a CAG-Cre transgenic mouse^51^. The genotypes of the resultant deletion mutant mice were checked by PCR (Supplementary Fig. 1c), and homozygotes and the heterozygotes in the C57BL/6 strain are maintained in NIG. The double knockout mouse of MRCS1 and MFCS4 was generated in NIG. To generate the double knockout alleles on the same chromosome, we established an ES cell line from blastocysts of the MRCS1 KO homozygotes. MFCS4 was then eliminated from the established MRCS1 KO ES cells using the same targeting construct as described in a previous study^21^. The chimeric mice were mated with C57BL/6. The double KO allele located in the *cis-*position was selected from alleles transmitted through the germline by genotyping. The heterozygotes in the C57BL/6 strain are maintained in NIG.

### Micro-CT analysis

X-ray micro computed tomography (micro-CT) analysis was carried out as previously described^52^. Briefly, embryos were soaked in a three-fold dilution of Lugol’s solution (2.5% potassium Iodideand 1.25% Iodine, WAKO) with deionized distilled water. The stained embryos were scanned using an X-ray micro-CT device (ScanXmate-E090S, Comscan Techno, Tokyo, Japan) at a tube voltage peak of 60 kVp and a tube current of 130 μA. Samples were rotated 360° in steps of 0.18°, generating 2,000 projection images of 992 × 992 pixels. The micro-CT data were reconstructed at an isotropic resolution of 5.3 × 5.3 × 5.3 μm. Three-dimensional tomographic images were obtained using the OsiriX program (www.osirix-viewer.com).

### Transgenic reporter assay

Enhancers for the transgenic assay were PCR-amplified from RP23-284A9 BAC DNA and the genome DNA derived from *Xenopus tropicalis*. Primer pairs for amplification of the enhancers are listed in Supplementary Table 2. Amplified DNA fragments were cloned into a construct including a *LacZ* reporter cassette with the *Hsp* promoter^21^. To abolish the LEF1 binding to motif1 of MRCS1, the potential LEF1 binding site at motif1 was mutated by changing GATGAAAG to GATGCACG. Injection of transgenes and X-gal staining were performed as described previously^21^.

### Nascent RNA detection

Antisense riboprobes that hybridize the intron 1 and 2 of the nascent RNA of *Shh* were synthesized as previously reported^53^. Briefly, two plasmid vectors containing the intron 1 and 2 of *Shh* were linearized with NotI and SpeI, respectively. RNA labeling was performed with digoxigenin RNA labeling mix (Roche) by *in vitro* transcription with T3 and T7 RNA polymerases (promega), respectively. The fragment size of the riboprobes was reduced to 500 bp or fewer by alkaline hydrolysis. Four micrometers paraffin sections of mouse embryos were deparaffinized in xylene. The sections were rehydrated and treated with 1 μg/ml Proteinase K. After dehydration through ethanol series, they were hybridized with riboprobes overnight at 65 °C. Monoclonal anti-Digoxigenin (Sigma) and Alexa488-conjugated anti-mouse IgG (Thermo Fisher Scientific) were used as primary and second antibodies. Nuclei were stained with 1 μg/ml of DAPI (Thermo Fisher Scientific).

### Electro mobility shift assay

HEK293T was transfected with the pcDNA3.1-*Lef1* using Lipofectamine 3000 (Invitrogen). After transfection, cells were incubated for one day and then harvested. Nuclear extracts were prepared by using CelLytic Nuclear Extraction Kit (Sigma). Complementary pairs of oligonucleotides were annealed and end-labeled with digoxigenin-conjugated ddUTP using DIG Gel Shift Kit, 2nd Generation (Roche). The probes were incubated with the nuclear extract for 15 minutes at room temperature, loaded onto a 6% native polyacrylamide gel and separated at 100 V for 1.5 hours. After electrophoresis, the probes were transferred to Hybond N+ membrane and the closslinked by UV irradiation. Detection of labeled probes was carried out using anti-digoxigenin-AP, Fab fragments (Roche) and CDP-star (Roche). Oligonucleotides used for making probes are shown in Supplementary Table 2.

### Luciferase assay

The 95 bp of core MRCS1 and 108 bp of MFCS4 fragments were PCR-amplified and cloned into the plasmid pGL4.23 (Promega) containing a firefly luciferase reporter. The plasmid pGL4.74 ubiquitously expressing *Renilla* luciferase was used for normalization. The protein-coding sequence of mouse *Lef1* was inserted into a mammalian expression vector, pcDNA3.1 (Life Technologies). HEK293T was maintained in Minimum Essential Medium (MEM) supplemented with 10% fetal bovine serum and 100 μg/ml penicillin–streptomycin. Cells at 70–90% confluency were transfected with pGL4.23 and PGL4.74 reporter plasmids, and with pcDNA3.1 using Lipofectamine 3000 (Invitrogen). Luciferase activity in the cell lysates was measured using a Dual-Luciferase Reporter Assay System (Promega) according to the manufacturer’s protocol.

## Electronic supplementary material

SHH signaling directed by two oral epithelium-specific enhancers controls tooth and oral development
