# Supplementary material for: SHH signaling directed by two oral epithelium-specific enhancers controls tooth and oral development
Source: Sci Rep. 2017 Oct 11;7:13004. doi: 10.1038/s41598-017-12532-y (PMC5636896; doi:10.1038/s41598-017-12532-y)
Supplement: Supplementary file 1 — SHH signaling directed by two oral epithelium-specific enhancers controls tooth and oral development [file 41598_2017_12532_MOESM1_ESM.pdf]

SHH signaling directed by two oral epithelium-specific enhancers controls tooth and oral development

Tomoko Sagai[1, #], Takanori Amano[1, #], Akiteru Maeno[1], Hiroshi Kiyonari[2], Hyejin Seo[3], Sung-Won Cho[3] and Toshihiko Shiroishi[1*]

[1]Mammalian Genetics Laboratory, National Institute of Genetics, Mishima, Shizuoka, Japan
[2]Animal Resource Development Unit and Genetic Engineering Team, RIKEN Center for Life Science Technologies, Kobe, Hyogo 650-0047, Japan.
[3]Division of Anatomy and Developmental Biology, Department of Oral Biology, Yonsei University College of Dentistry, Seoul, Korea

#These two authors contributed equally to this study.
*Author for correspondence: Toshihiko Shiroishi

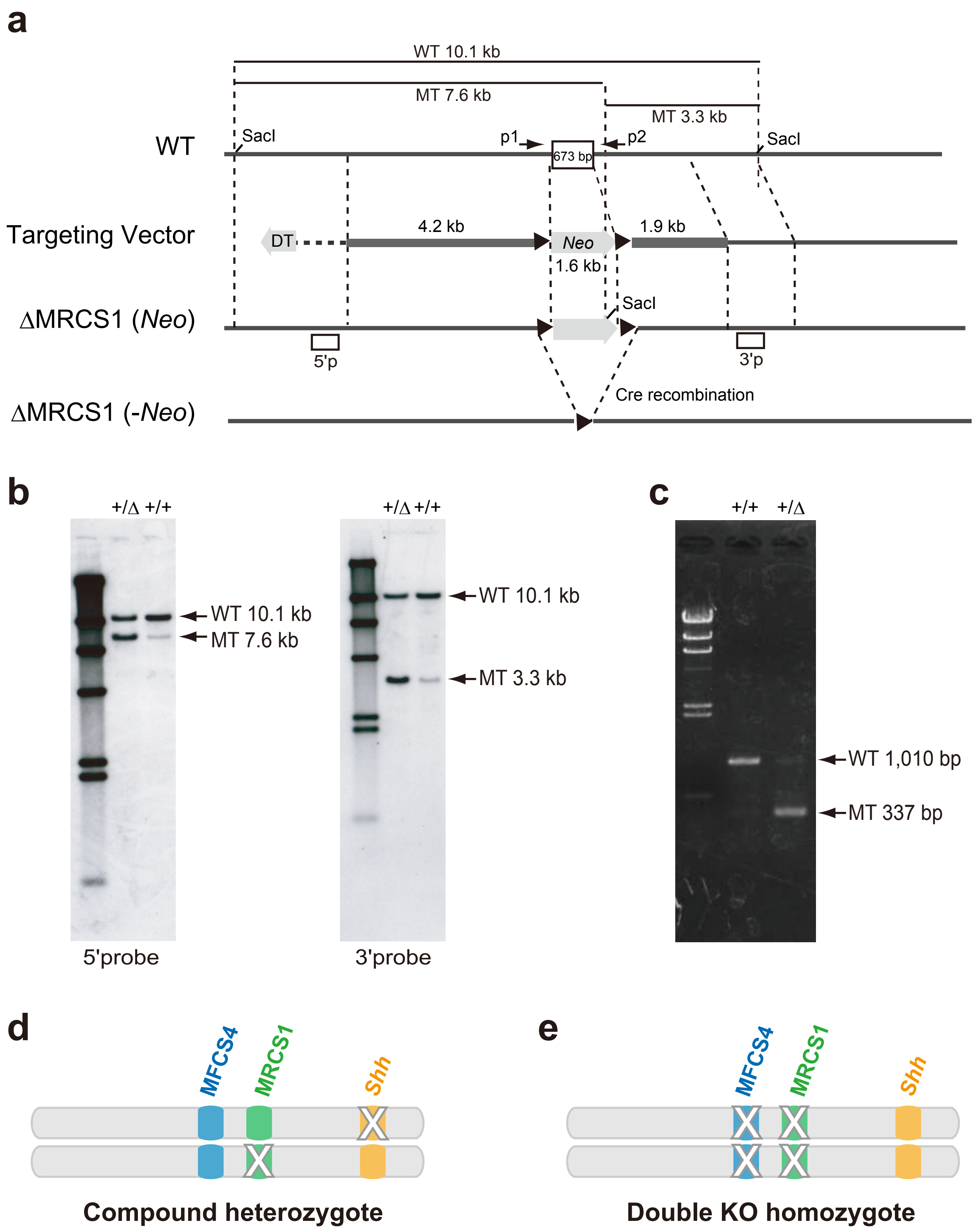

Supplementary Fig. 1. Generation of mutant mice in this study. (a) Schematic diagram of the ES cell targeting of MRCS1. (b) Southern blot analysis was carried out for SacI-digested genomic DNAs. The 5' probe (5' p in (a)) detected fragments of 10.1 kb in the wild type allele (WT) and 7.6 kb in the mutant allele (MT). The 3' probe (3' p in (a)) detected fragments of 10.1 kb in WT and 3.3 kb in MT. (c) PCR amplification of the WT allele (1,010 bp) and the MT allele (337 bp) with a primer pairs p1-p2. The left lanes are λ/HindIII marker. Schematic diagram of the mouse chromosome 5 illustraing composition of mutant alleles in the compound heterozygote of the MRCS1 KO and the Shh coding sequence KO alleles (d), and the homozygote of the double KO mouse of MRCS1 and MFCS4 (e).

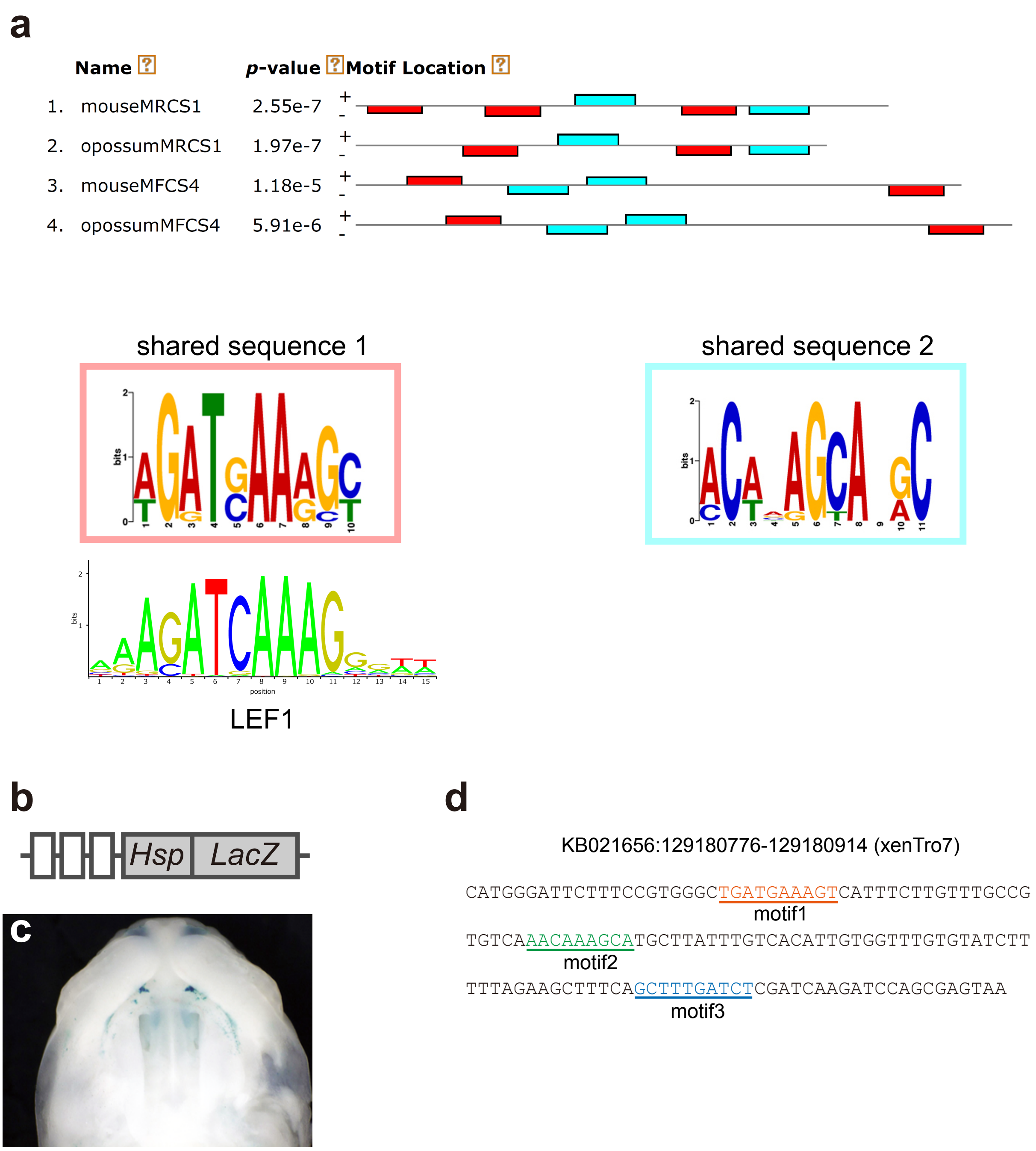

Supplementary Fig. 2. Sequences shared between MRCS1 and MFCS4. (a) Shared short sequences in MRCS1 and MFCS4 of mouse and opossum were identified by MEME (red and blue boxes). Position weight matrix of the two shared sequences and LEF1 binding sites are shown. (b) The *LacZ* reporter transgene construct with three tandem repeats. (c) The maxillary occlusal view of the E13.5 mouse embryo. The *LacZ* expression driven by the three tandem copies of the core sequence of mouse MRCS1 was observed in the incisor and molar rudiments but not in the palatal rugae. (d) The three shared motifs in the core sequence of *Xenopus* MFCS4.

Supplementary Table1 Incidence of the supernumerary tooth

Genotype	Mice with Maxillary sn		Mice with Mandibular sn		Incidence
	Right	Left	Right	Left	
wild type	0	0	0	0	0/20 (0%)
∆MRCS1/+	0	0	0	0	0/12 (0%)
*Shh*KO/+	0	0	0	0	0/12 (0%)
∆MRCS1/∆MRCS1	0	1	1	1	1/21 (4.7%)
∆MRCS1/*Shh*KO	8	6	10	12	15/19 (78.9%)
∆MFCS4/∆MFCS4	0	0	0	0	0/15 (0%)
DKO/DKO	4	3	6	5	8/12 (66.7%)

sn, supernumerary tooth. Incidence is indicated as affected mice/observed mice.

Supplementary Table1 Incidence of the supernumerary tooth

Genotype	Mice with Maxillary sn		Mice with Mandibular sn		Incidence
	Right	Left	Right	Left	
wild type	0	0	0	0	0/20 (0%)
ΔMRCS1/+	0	0	0	0	0/12 (0%)
*Shh*KO/+	0	0	0	0	0/12 (0%)
ΔMRCS1/ΔMRCS1	0	1	1	1	1/21 (4.7%)
ΔMRCS1/*Shh*KO	8	6	10	12	15/19 (78.9%)
ΔMFCS4/ΔMFCS4	0	0	0	0	0/15 (0%)
DKO/DKO	4	3	6	5	8/12 (66.7%)

sn, supernumerary tooth. Incidence is indicated as affected mice/observed mice.

Supplementary Table 2 Primers and oligonucleotides used in this study

**For ESC targeting**		
Long arm	F	AGGAACTTTCAGTCTCACTGGG
	R	AATCTCGAGCTTGTGGGTTTGTCTCCTTG
Short arm	F	TAAGGATCCCAGAGAGTGACTGCCAAATG
	R	TTACCCGGGTATGCTCTCTTGAGCCTAGG
**Probes for Southern blotting**		
5' probe	F	TGCCTTATTAGATCTTGATC
	R	CTATGCTCTCCTGCACTGTT
3' probe	F	AGGCCAGAGCCCAGAACCTA
	R	GATGAGGTCAGAGGATGACT
**For genotyping**		
P1	F	CATTGCCACAGAGCAGAAGT
P2	R	TGTGCACCTCTATGTGTGCC
**For cloning enhancers**		
Mouse whole MRCS1	F	AGGAGACAAACCCACAAGAG
	R	CATTTGGCAGTCACTCTCTG
Mouse core MRCS1	F	AAGGCTTCACCTGCTTTTGC
	R	CCTTTAATACCGGTCCTGAG
Xenopus whole MFCS4	F	TGTGTATCAGGAAGCAGAGC
	R	AAGACAGAACATGGGCATGG
Xenopus core MFCS4	F	CCTGCAGACAACACAATTAG
	R	GTGACAATATACATCCCTGG
Mouse whole MFCS4	F	AGCAGGTTAAACCGCAGTTC
	R	TCCATCCCAGCTTCTAATGC
Mouse core MFCS4	F	TAATCAGGCTGATGAAAGTC
	R	TTTAGATCAAAGCGCAAAAC
**For deletion constructs**		
del1	F	CTGCCAAACAACAAAGCTT
	R	CATTTGGCAGTCACTCTCTG
del2	F	AGGAGACAAACCCACAAGAG

	R	CCTCTTGGTTTTCCTTTGCT
del3	F	AGGAGACAAACCCACAAGAG
	R	AAGGCTTCACCTGCTTTTGC
del4	F	AGGAGACAAACCCACAAGAG
	R	CTCAGGACCGGTATTAAAGG
Δmo1 (inverse PCR)	F	CTTTGCAGACAAAGCAGGCT
	R	CCGGGCAAAAGCAGGTGAAG
Δmo2 (inverse PCR)	F	GGCTGGGTTGGGCCTTGATC
	R	TGCAAAGATGAAAGCCGGGC
Δmo3 (inverse PCR)	F	AGGGCGTGCTCAGGACCGGT
	R	GCCCAACCCAGCCTGCTTTG
mut mo1 (inverse PCR)	F	TGCATCTTTGCAGACAAAGCAGGCTGGGTTG
	R	CGCCGGGCAAAAGCAGGTGAAGCCTTTTTTG
**Oligonucleotides for EMSA**		
MRCS1-motif1-WT	S	CCTGCTTTTGCCCGGCTTTCATCTTTGCAGACAAAGC
	A	GCCTGCTTTGTCTGCAAAGATGAAAGCCGGGCAAAAG
MRCS1-motif1-mut	S	CCTGCTTTTGCCCGGCgTgCATCTTTGCAGACAAAGC
	A	GCCTGCTTTGTCTGCAAAGATGcAcGCCGGGCAAAAG
MFCS4-motif1-WT	S	ATTCTTAATCAGGCTGATGAAAGTCATTTATTGTTTG
	A	ACAGCAAACAATAAATGACTTTCATCAGCCTGATTAA
MFCS4-motif1-mut	S	ATTCTTAATCAGGCTGATGcAcGTCATTTATTGTTTG
	A	ACAGCAAACAATAAATGACgTgCATCAGCCTGATTAA

* F, forward primer; R, reverse primer; S, sense oligonucleotide; A, antisense oligonucleotide.
